# A systematic scoping review of the genetic ancestry of the Brazilian population

**DOI:** 10.1590/1678-4685-GMB-2018-0076

**Published:** 2019-11-14

**Authors:** Aracele Maria de Souza, Sarah Stela Resende, Taís Nóbrega de Sousa, Cristiana Ferreira Alves de Brito

**Affiliations:** 1 Research Group in Molecular Biology and Immunology of Malaria, Instituto René Rachou, Fiocruz, Belo Horizonte, MG, Brazil.

**Keywords:** Scoping review, genetic ancestry, Brazilian population, genetic admixture

## Abstract

The genetic background of the Brazilian population is mainly characterized by three parental populations: European, African, and Native American. The aim of this study was to overview the genetic ancestry estimates for different Brazilian geographic regions and analyze factors involved in these estimates. In this systematic scoping review were included 51 studies, comprehending 81 populations of 19 states from five regions of Brazil. To reduce the potential of bias from studies with different sampling methods, we calculated the mean genetic ancestry weighted by the number of individuals. The weighted mean proportions of European, African, and Native American ancestries were 68.1%, 19.6%, and 11.6%, respectively. At the regional level, the highest European contribution occurred in the South, while the highest African and Native American contributions occurred in the Northeastern and Northern regions, respectively. Among states in the Northeast region, Bahia and Ceará showed significant differences, suggesting distinct demographic histories. This review contributes for a broader understanding of the Brazilian ancestry and indicates that the ancestry estimates are influenced by the type of molecular marker and the sampling method.

## Introduction

The Brazilian population has one of the most heterogeneous genetic constitutions in the world with a predominant tri-hybrid composition and an extensive admixture (Sans, 2000). This admixture is the result of more than 500 years of interethnic crosses between Native Americans, European colonizers, and African slaves. The study of the Native Americans genetic history is still quite controversial. Recent surveys of genetic diversity in Native Americans replaced the hypothesis of a single stream of migration to a complex scenario involving multiple sources (Reich *et al.*, 2012). Most of Native American populations derived from the “First American” ancestral population, which crossed the Bering Strait more than 15,000 years ago (Tamm *et al.*, 2007; Kitchen *et al.*, 2008; Fagundes *et al.*, 2008). However, other ancestral population streams were also detected with extensive gene flow among them (Lell *et al.*, 2002; de Azevedo *et al.*, 2011; Reich *et al.*, 2012).

The estimated number of Native Americans living in Brazil was around 2.5 million when the first Portuguese colonizers arrived (Salzano and Freire-Maia, 1970; Bethell, 1999). The admixture, predominantly between European men and Native American women started immediately, however, conflicts and diseases contributed to a drastic reduction in the Native American population (Salzano and Freire-Maia 1967, 1970; Ribeiro, 1995). African people were introduced in Brazil as slaves for sugarcane farms and later for the gold and diamond mines and coffee plantations. During slave trade (1452 - 1870), it is estimated that around 4 million Africans arrived in Brazil, mainly from Guinea, Congo, Angola, Mozambique, and Nigeria (Salzano and Freire-Maia, 1967; Curtin, 1969; Ribeiro, 1995; Salzano and Bortolini, 2002). During colonization, more than 500,000 Portuguese people arrived in Brazil; when the ports were legally open, Italian, Spanish, and German also came to Brazil. Asian and Middle Eastern immigration started only in the 20th century, mainly from Japan, but also from Lebanon and Syria (Salzano and Freire-Maia, 1967). From 1500 to 1972, the immigrants to Brazil were 58% Europeans, 40% Africans, and only 2% Asian (Callegari-Jacques and Salzano, 1999).

Although the biological formation of the Brazilian people is due to the contribution of Native Americans, Europeans, and Africans, there may be a greater relative influence of one or another group depending on the geographical region. Variation in the process of colonization and occupation of the Brazilian regions created a diverse degree and extent of genetic admixture across the country. To understand the differences in the admixture among Brazilian individuals and estimate the contribution of these ancestral groups in populations from different geographical regions, several studies have been carried out. These studies have been performed using distinct panels of molecular markers, such as mitochondrial DNA (mtDNA) (Alves-Silva *et al.*, 2000), Y-chromosome (Santos *et al.*, 1996), short tandem repeats (STRs) (Callegari-Jacques *et al.*, 2003), insertion/deletions (INDELs) (Santos *et al.*, 2010), and single nucleotide polymorphisms (SNPs) (Lins *et al.*, 2011a). Ancestry-informative markers (AIMs) are SNPs and INDELs selected to maximize the absolute difference in allele frequency between two ancestral populations, therefore they are powerful tools for inferring the genetic composition of admixed populations (Enoch *et al.*, 2006; Kosoy *et al.*, 2009). More recently, the development of high throughput genotyping technologies allowed genome-wide studies to infer the genomic ancestry using a large number of SNPs (Kehdy *et al.*, 2015; Mychaleckyj *et al.*, 2017). Studies using matrilineages (mitochondrial DNA) and patrilineages (Y-chromosome) reveal that in Brazil an asymmetric genetic sex-biased admixture has occurred, where males have, in general, higher European ancestry and females have higher African or Native American ancestry (Alves-Silva *et al.*, 2000; Carvalho-Silva *et al.*, 2001).

Using a set of 40 INDEL polymorphisms, Pena *et al.* (2011) demonstrated that genetic ancestry among Brazilian regions is more homogenous than previously expected, particularly concerning the high European ancestry observed in all regions. However, several studies have been highlighting the heterogeneous aspects of Brazilian regions. For instance, a large community-based study was carried out in three cities that represent different regions of Brazil and different scenarios of genetic structure (size, kinship, and inbreeding) (Kehdy *et al.*, 2015). Another example is a study performed by Manta *et al.* (2013a) that included populations from all five Brazilian regions. In this scoping review, we conducted a robust review of studies estimating the contribution of the three parental populations (European, African, and Native American) in the genetic makeup of the Brazilian population. Here, we selected studies of 81 populations from different Brazilian localities for which genetic ancestry was estimated using nuclear autosomal makers. Considering the difficulty of real random sampling and the consequent potential bias introduced by studies with very different sample sizes, we estimated the genetic ancestry weighted by the number of individuals in each study. Moreover, we also analyzed other factors influencing the genetic ancestry estimates, such as type and number of molecular markers. Therefore, this review allows a more comprehensive understanding of the genetic ancestry estimation across the country and how the sampling and panel of markers have contributed to the current estimated pattern of admixture among Brazilian regions.

## Materials and Methods

### Study design

This study was based on Arksey and O’Malley’s scoping review framework (Arksey and O’Malley, 2005). The key steps followed were:

- Identifying the research questions: What are the estimates of the genetic ancestry for Brazilian populations from different regions? How is the estimate of genetic ancestry affected by the panel of markers and sampling methods used for its identification?
- Identifying relevant studies: The survey was performed based on articles published in two electronic databases/portals, PubMed and BVS (MEDLINE, LILACS, HISA, BBO, Coleciona SUS, Sec. Est. Saúde SP, IBECS, INDEX Psicologia) until November 1^st^ 2017. The search terms used are shown in Table S1.
- Study selection: Two members of our research group independently screened all retrieved titles, abstracts, or full-text articles for applicability to the review’s research questions. All citations were imported into an Excel worksheet and duplicate citations were manually removed. Articles were eligible if they met the inclusion criteria. Discrepancies resulting from the independent initial screening were discussed by the two research team members until consensus was reached. Articles that could not be obtained as full-text through online databases were also excluded from final analysis.
- Charting the data: The data were entered onto a ‘data charting form’ using the Excel software to record key information from the included studies. The extracted data included the following sections: manuscript title, origin of human samples (region, state, and city, if available), number of individuals, number of markers, type of genetic markers, date of samples collection, and estimated percentage of European, African, and Native American ancestries.
- Collecting, summarizing, and reporting the results - this process involved descriptive analyzes of all included studies.

### Inclusion/exclusion criteria

Articles found in databases were examined for the following inclusion/exclusion criteria. Inclusion criteria: papers published until October 2017; studies using autosomal molecular markers to infer on genetic ancestry; studies that estimate European, African, and Native American genetic ancestries; studies that provided the number of individuals studied by each Brazilian State. Exclusion criteria: duplicated studies; full-text unavailable; studies based on individuals from other countries; studies using non-human samples; review article; other text formats different from research articles and theses; non-original articles describing the ancestry of the population estimated by others; without information about the state of origin of the sample individuals; lacking data on ancestry for the three ancestral populations (European, African, and Native American); case or family descriptions; studies based on related individuals; studies using blood group or uniparental markers; studies using only specific ethnic groups (for example, Afro-descendants or Caucasians). Studies with partially isolated populations, such as Native American tribes and Afro-descendants’ communities (Quilombos) were analyzed separately.

### Statistical analysis

Data from the descriptive analyzes of the ancestry are presented as mean and standard deviation. The mean ancestry in cities, states, and regions and /or the molecular markers were weighted by the number of individuals included in each study by using Minitab 18.1 (State College, PA, USA).

Kruskal-Wallis test was performed and, if significance was found, Dunn’s multiple comparisons post-hoc test was used to define the differences among ancestry estimates based on type of molecular markers (INDEL, SNP, and STR) and to verify differences in ancestral estimates among regions and states. Comparisons among Brazilian regions and partially isolated afro-descendant communities were performed using Mann-Whitney U test. Correlation analysis (Spearman’s Rho test or Fischer’s test) was performed to verify associations between number of individuals or numbers of molecular markers and European, African, and Native American parental ancestries for each city. A *p*-value < 0.05 was considered significant. All statistical analyses were done using GraphPad Prism 5.0 (San Diego, California, USA) and Minitab 18.1. Triangular plots of the proportions of European, African, and Native American genetic ancestries were obtained using the Tri-Plot program (Graham and Midgley, 2000).

## Results

### Summary of included studies

**Figure 1:**
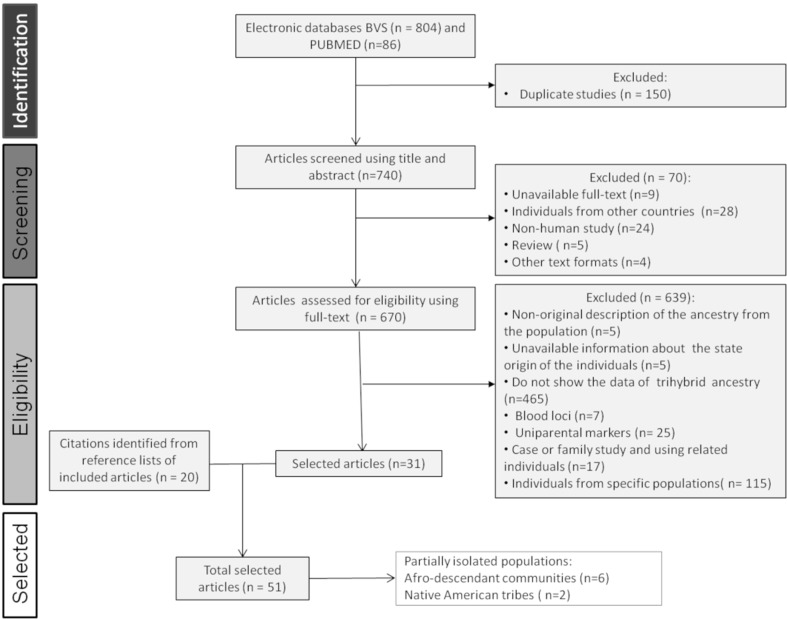
Flowchart of the literature search and screening process executed in this scoping review.

The scoping review search included papers published from 2002 to 2017. A total of 890 studies was identified in the electronic databases BVS (n = 804) and Pubmed (n= 86) with the selected search terms (Table S1; Figure 1). After removing all duplicated documents, a total of 740 titles and abstracts were screened. From these, 70 studies were excluded (full-text not available; sample individuals were from countries other than Brazil; studies based on non-human samples; article’s format different than research papers). Finally, 670 full-text articles were assessed in detail for eligibility. Of these, 639 publications were excluded due to not completely fulfilling our inclusion criteria. We decided to analyze only studies of ancestry estimates based on autosomal markers and to exclude studies using classical markers or uniparental markers. As a result, 51 studies were selected, 31 identified by scoping review and 20 added from the references of the selected articles, comprehending a total of 81 populations (Table S2). Moreover, the partially isolated populations, Native American tribes, and Afro-descendant communities were analyzed separately (Table S3).

### Ancestry by Brazilian geographic region

**Figure 2:**
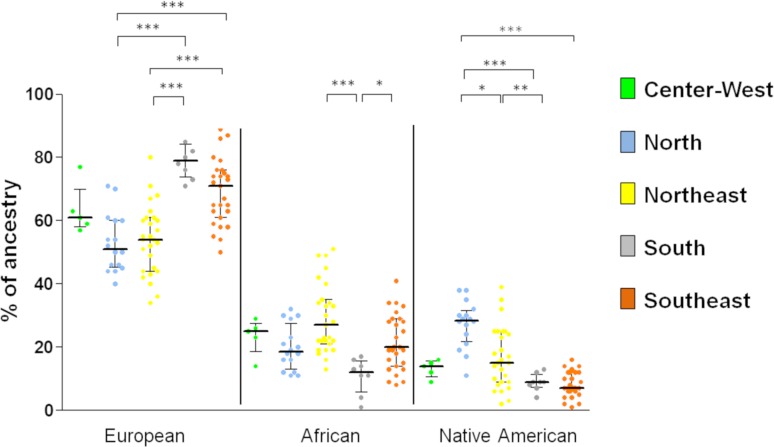
Genetic ancestral estimates of studies from different geographic regions. Asterisks indicate the statistical differences (Kruskal-Wallis test, * *p* < 0.01; ** *p* < 0.001, *** *p* < 0.0001).

The genetic ancestry estimates of Brazilian populations identified in this review comprehended 19 states from the five political geographic regions (Table 1). All regions showed a higher prevalence of European ancestry (mean 62.4% for Brazil), which was significantly higher in the South and Southeast regions compared to the North and Northeast regions (Figure 2). African ancestry was the second most prevalent ancestral population in Brazil (22.6%) and was significantly lower in the South compared to Northeast and Southeast regions. Native American ancestry was the less prevalent in the Brazilian population (14.7%), and was significantly higher in the North region compared to the Northeast, South, and Southeast regions (Figure 2). The South region showed the most distinct profile of ancestry, with the highest number of significant differences compared to the other regions of the country.

**Table 1:** Characterization of the ancestry profile of Brazilian geographical regions based on all 51 manuscripts included in the scoping review.

Geographical region	State	City	N	Mean± SD			Weighted Mean± weighted SD			Ref
				EUR	AFR	AME	EUR	AFR	AME	
Center-West	Distrito Federal	Brasilia	780	65.0± 10.58	22.0± 7.55	13.0± 3.61	63.0± 10.26	23.9± 7.65	13.1± 3.13	1 - 3
		Taguatinga#	189	63.0	25.0	12.0	63.0	25.0	12.0	4
		Total/Mean of the state	969	64.5± 8.70	22.8± 6.34	12.7± 2.98	63.0± 8.67	24.1± 6.49	12.9± 2.70	
	Mato Grosso do sul	NI#	84	59.0	26.0	15.0	59.0	26.0	15.0	5
		Total/mean of the state	84	59.0	26.0	15.0	59.0	26.0	15.0	
	Total/mean of the region		1053	63.4± 7.92	23.4± 5.68	13.2± 2.78	62.7± 8.15	24.2± 6.06	13.1± 2.59	
North	Pará	Belém	1044	54.5± 10.19	18.33± 7.00	27.17± 4.17	55.5± 10.43	17.60± 7.06	26.9± 4.44	6 - 11
		Santarém#	90	60.0	11.0	29.0	60.0	11.0	29.0	13
		Goianésia do Pará#	273	44.0	32.0	25.0	44.0	32.0	25.0	14
		NI#	210	45	23	32	45	23	32	12
		Total/mean of the state	1617	52.9± 9.53	19.6± 7.86	27.56± 3.90	52.4± 9.88	20.4± 8.24	27.2± 4.18	
	Amapá	Macapá	437	48.0± 2.82	24.0± 7.07	28.0± 9.89	47.2± 2.59	22.0± 6.46	30.8± 9.05	15-16
		Total/mean of the state	437	48± 2.82	24± 7.07	28± 9.89	47.2± 2.59	22.0± 6.46	30.8± 9.05	
	Amazonas	Manaus	294	58.5± 17.68	14.0± 2.82	27.5± 14.85	67.4± 12.37	12.6± 1.98	20.0± 10.39	5,17
		Rio Pardo#	340	44.0	18.0	38.0	44.0	18.0	38.0	18
		Total/mean of the state	634	53.7± 15.04	15.3± 3.05	31.0± 12.12	54.9± 16.06	15.5± 3.51	29.6± 12.6	
	Rondônia	Porto Velho	404	57.0± 4.24	24.0± 8.48	19.0± 12.02	55.8± 3.89	21.6± 7.79	22.9± 11.04	19-20
		Total/mean of the state	404	57.0± 4.25	24.0± 8.49	19.0± 12.03	55.8± 3.89	21.6± 7.79	22.9± 11.04	
	Total/mean of the region		3092	52.9± 9.27	19.9± 7.14	27.3± 7.41	52.6± 9.72	19.8± 6.95	27.7± 7.22	
Northeast	Ceará	Barbalha#	60	68.0	19.0	13.0	68.0	19.0	13.0	13
		Fortaleza	1712	55.4± 7.13	19.9± 3.8	24.7± 5.55	54.0± 7.33	18.7± 3.81	27.1± 7.00	21-22
		Total/mean of the state	1772	57.0± 7.96	19.8± 3.53	23.2± 6.60	54.5± 7.63	18.7± 3.71	26.7± 7.34	
	Bahia	Salvador	4859	52.6± 14.15	39.8± 11.19	7.5± 4.11	47.9± 12.42	43.8± 9.21	8.3± 4.80	21,23-28
		Ilheus	256	61± 0.00	31.5± 2.12	7.5± 2.12	61± 0.00	31.3± 2.10	7.7± 2.10	7,29
		Jequie#	20	44.0	42.0	11.0	44.0	42.0	11.0	30
		Total/mean of the state	5135	53.4± 12.69	38.6± 10.03	7.8± 3.66	48.5± 12.23	43.18± 9.25	8.29± 4.60	
	Alagoas	Maceió	120	67	19	14	67	19	14	5,13
		NI#	104	55	27	19	55	27	19	
		Total/mean of the state	224	61.0± 8.49	23.0± 5.66	16.5± 3.55	61.4± 8.46	22.7± 5.64	16.3± 3.52	
	Maranhão	São Luis#	177	42.0	19.0	39.0	42.0	19.0	39.0	31
		Total/mean of the state	177	42.0	19.0	39.0	42.0	19.0	39.0	
	Piauí	NI#	204	60.0	22.0	18.0	60.0	22.0	18.0	32
		Total/mean of the state	204	60.0	22.0	18.0	60.0	22.0	18.0	
	Pernambuco	Recife#	192	60.0	23.0	17.0	60.0	23.0	17.0	33
		NI#	133	57.0	28.0	15.0	57.0	28.0	15.0	5
		Total/mean of the state	325	58.5± 2.12	25.5± 3.54	16± 1.41	58.8± 2.08	25.0± 3.47	16.2± 1.39	
	Total/mean of the region		7837	55.3± 10.16	28.8± 11.31	15.8± 9.45	50.8± 11.09	35.2± 13.56	13.9± 9.99	
South	Rio Grande do Sul	Porto Alegre	7197	81.7± 3.51	9.3± 4.72	8.3± 1.15	84.7± 1.49	4.5± 2.27	8.9± 0.43	7,17,34
		Pelotas#	3736	76.0	16.0	8.0	76.0	16.0	8.0	25
		NI#	81	95	1	4	95	1	4	9
		NI#	23	73	14	13	73	14	13	5
		Total/mean of the state	11037	81.5± 7.86	9.8± 5.98	8.3± 2.94	81.8± 4.82	8.4± 6.23	8.6± 0.75	
	Paraná	NI#	21	71.0	17.0	12.0	71.0	17.0	12.0	5
		Total/mean of the state	21	71.0	17.0	12.0	71.0	17.0	12.0	
	Santa Catarina	NI#	20	80.0	11.0	9.0	80.0	11.0	9.0	5
		Total/mean of the state	20	80.0	11.0	9.0	80.0	11.0	9.0	
	Total/mean of the region		11078	80.0± 7.60	10.9± 5.64	8.9± 2.80	81.8± 4.72	8.4± 6.08	8.6± 0.74	
Southeast	Espirito Santo	NI#	92	74	13	13	74	13	13	5
		Total/mean of the state	92	74	13	13	74	13	13	
	Minas Gerais	Belo Horizonte	324	70.5± 7.77	24.5± 14.14	5.5± 6.36	68.0± 6.97	28.7± 12.03	3.5± 5.70	35-36
		Alfenas	758	88.0± 1.41	10.0± 0.70	2.0± 0.70	87.8± 1.38	10.2± 1.38	2.0± 0	23,37
		Manhuaçu#	30	73	19	8	73	19	8	39
		Montes Claros#	24	73	19	8	73	19	8	39
		Ouro Preto#	189	52	38	10	52	38	10	40
		Bambui#	1442	79	14	7	79	14	7	26
		NI#	88	59	29	12	59	29	12	5
		NI#	291	58	34	4	58	34	4	38
		Total/mean of the state	3146	68.0± 13.83	24.7± 11.42	6.8± 4.92	75.4± 12.04	18.3± 9.82	5.8± 3.95	
	São Paulo	Araraquara#	403	76	18	12	76	18	12	41
		São Paulo	2346	67.0± 8.4	21.8± 8.9	9.2± 3.11	68.0± 8.71	22.5± 7.08	8.8± 3.46	37,42-45
		Botucatu	983	73.5± 9.19	12.0± 5.65	9.0± 3.53	74.8± 8.99	11.2± 5.53	8.9± 3.46	46-47
		Campinas#	109	73	20	7	73	20	7	17
		Ribeirão Preto#	448	86	9	4	86	9	4	48
		NI#	49	63	25	12	63	25	12	5
		Total/mean of the state	4338	70.9± 8.79	18.6± 7.90	8.54± 3.11	72.5± 9.08	18.1± 7.84	8.1± 3.20	
	Rio de Janeiro	Rio de Janeiro	880	67.0± 9.05	23.3± 5.44	10± 3.56	63.9± 10.20	25.3± 6.39	10.9± 3.78	7,13,49,50
		NI#	335	65	28	7	65	28	7	51
	Total/mean of the state		1215	66.6± 7.89	24.2± 5.17	9.4± 3.36	64.2± 8.42	26.0± 5.44	9.9± 3.69	
	Total/mean of the region		8791	69.1± 10.46	21.7± 9.21	8.2± 3.97	72.3± 10.39	19.2± 8.49	7.6± 3.73	
Total/mean of the country			31851	62.4± 13.07	22.6± 10.37	14.7± 9.63	68.1± 15.68	19.6± 13.64	11.6± 8.20	

**Figure 3:**
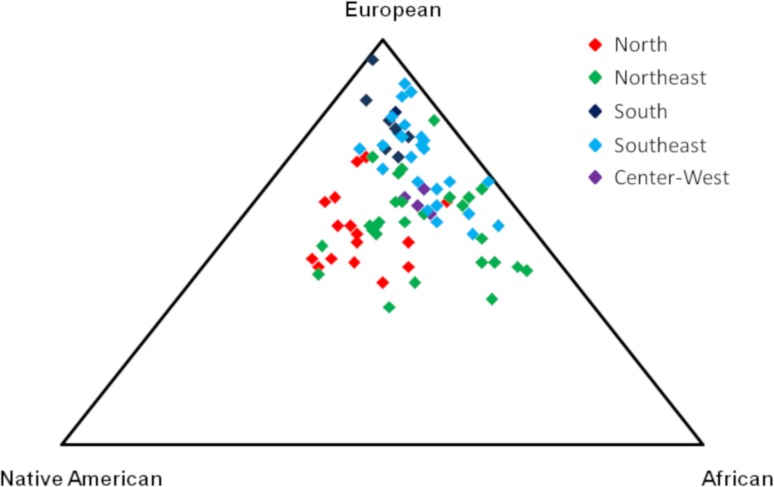
Tri-plot showing the clustering of the genetic ancestry inferred by studies from different regions of Brazil. Populations of the Center-West are represented in purple, North in red, Northeast in green, South in dark blue, and Southeast in light blue.

Triangular plot analysis was performed to visualize the spatial distribution of the populations from different Brazilian regions according to their ancestries. The triangular plot revealed that all populations are close to the European vertex (Figure 3). The populations of the South and Southeast regions are the closest to the European vertex. The Northeast populations showed a shift to the African and Native American ancestry vertices. The North populations showed more proximity to Native American ancestry vertex. The center-west populations showed an intermediate position overlapping with Southeast and Northeast population.

### Local differences in ancestry estimates for Brazilian populations

**Figure 4:**
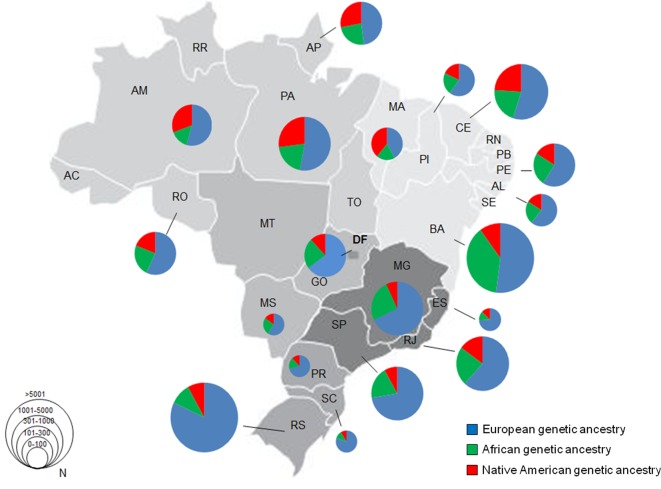
Ancestral profiles of Brazilian states according to the scoping review. The graphs on the map represent the median of ancestry in Brazilian regions. The different sizes of the graphs reflect the sample sizes (scheme on the left).

Next, we evaluated the differences in ancestry estimates among populations of the different Brazilian states (Figure 4). Higher prevalence of European ancestry was observed for populations from all Brazilian states, and the highest was identified in Rio Grande do Sul (RS) (81.5%). On the contrary, the Maranhão state (MA) showed the lowest percentage of European ancestry (42.0%). Bahia (BA) showed the highest percentage of African ancestry (38.6%), while the lowest percentage was observed in Rio Grande do Sul (9.8%). Native American ancestry was highest in Maranhão (AM) (39%) and lowest in Minas Gerais state (MG) (6.8%).

Comparing the states from each region, significant differences were observed only in the Northeast region (Figure S1). The African ancestry was significantly higher in Bahia (BA) than in Ceará (CE) (Kruskal-Wallis test, *p*=0.004) and the Native American ancestry was significantly lower in Bahia than in Ceará (Kruskal-Wallis test, *p*=0.001).

### Influence of molecular markers in the genetic ancestry estimates

Studies were subdivided into three main categories according to the type of markers used in the analysis: those using INDELs, SNPs, or STR/VNTRs (Table 2). Most studies used INDELs (23 articles), followed by SNPs (15 articles), and STR (7 articles) to estimate genetic ancestry of Brazilian populations. The number of markers used in each study was highly variable, particularly for SNPs (ranging from 8 to 331,790). Comparisons of ancestry estimates for the same geographic region, but accessing different samples of individuals and using different molecular markers showed that only for Northeast region the European ancestry estimate was different using INDELs and SNPs (Table 2). The ancestry estimates using different molecular markers were also compared in studies sampling different individuals in the same city (Belém/PA, Macapá/AP, Manaus/AM, Porto Velho/RO, Fortaleza/CE, Salvador/BA, Porto Alegre/RS, São Paulo/SP and Belo Horizonte/MG) (Table S2). For cities with more than one study for each type of marker, the comparison was performed using the mean. Significant differences (Chi-square test or Fisher’s test, *p* < 0.05) in ancestry estimates concerning molecular markers of distinct types were observed for Belem, Manaus, Porto Velho, Fortaleza, and Belo Horizonte (Table S2). Comparison of ancestry estimates of the same sample of individuals using different markers was possible for two studies, Teló (2010) and Pedrosa (2006). Statistically significant differences were observed for a study in Salvador comparing STR and INDEL (Table S2, ref 26), and for Afro-descendant communities comparing STR, SNP/INDEL, and a combination of both (Table S3, ref 52).

**Table 2:** Description of molecular marker panels used to estimate the genetic ancestry in the selected studies from different Brazilian regions.

Geographic region	Number of samples	Type of marker	Number of markers	Mean of Ancestry						REFERENCES
				EUR	EUR (weighted)	AFR	AFR (weighted)	AME	AME (weighted)	
CW	84	INDEL	46	59	NA	26	NA	15	NA	5
	801	SNP	12 - 28	66	63.4	23	24.3	12	12.3	1,3,4
N	1622	INDEL	40 - 48	53	53.3	19	20.0	28	26.7	5, 7,8,9,10,11,12,13
	507	SNP	8 - 48	42	42.7	24	22.0	30	35.4	6, 18
	681	STR	4 - 12	59	57.8	20	18.4	21	24.0	16,17,19
NE	2245	INDEL	9 - 46	63*	62.9	25	28.3	11	8.9	5, 7, 13, 23, 24, 26 ,32
	3528	SNP	8 - 331,790	49*	48.7	29	34.5	21	16.7	21, 22, 25, 30, 33
	380	STR	4 - 8	51	51.1	26	26.5	24	22.4	26, 31
S	7116	INDEL	40 - 48	80	84.8	10	4.3	9	8.9	5, 7, 9, 34
	3736	SNP	331,790	76	NA	16	NA	8	NA	25
	226	STR	12	82	NA	11	NA	7	NA	17
SE	4552	INDEL	40 - 61	71	72.7	19	17.8	9	8.4	5, 7, 13, 23, 36, 37, 44, 47, 48, 49, 50, 51
	3202	SNP	14 - 331,790	65	73.4	25	18.5	8	7.1	25, 39, 40, 42, 43,45
	746	STR	18 - 34	71	72.1	24	23.3	5	4.6	17, 35, 41

The association between the number of markers and ancestry estimates was not statistically significant for studies from the same city (data not shown). In this analysis, studies using genome-wide SNPs were excluded, thus analysis was performed for studies using samples from Belém, Salvador, Porto Alegre, São Paulo, and Rio de Janeiro.

### Influence of sampling in the genetic ancestry estimates

To balance the ancestry estimates based on studies with different number of samples, we calculated a weighted mean. A slightly higher proportion of European ancestry (weighted mean 68.1%, mean 62.4%) and lower African (weighted mean 19.6%, mean 22.6%) and Native American ancestries (weighted mean 11.6%, mean 14.7%) were observed (Table 1). However, these differences between mean and weighted mean were not statistically significant (Mann Whitney, *p*=0.965 for European ancestry, *p*=0.974 for African ancestry, and *p*=0.990 for Native American ancestry). Concerning regions, the weighted mean showed also slightly different results, particularly for European and African ancestries for the Northeast region (Table 1).

Correlation analysis between number of individuals and ancestry estimated for each geographic region using the same type of marker did not show any significant result (Spearman’s correlation test, *p* > 0.05). Moreover, the analysis comparing different number of individuals from the same city was performed for Belem/PA, Salvador/BA, and Rio de Janeiro/RJ by INDEL; and Fortaleza/CE and São Paulo/SP by SNP ancestry estimates. No significant result was observed (data not shown).

### Ancestry estimates among Afro-descendant communities and Native American tribes

Concerning studies of partially isolated ethnic populations included in this review, two focused in Native American tribes (Terena/MS and Santa Isabel/AM) and 15 in Afro-descendant communities from different geographical Brazilian regions (Table S3). The two Native American tribes were studied by the same group, using the same panel of molecular markers and showed similar results of estimated ancestry (mean of 15% European, 8% African, and 77% Native American), despite the tribes being from different geographic regions. Among the studies that analyzed the Afro-descendant populations, the ancestry estimates were quite variable with the means of 32.7% (ranging from 4 to 74%) for European ancestry, 52.4% (26 to 89%) for African, and 14.8% (0 to 38%) for Native American. The ancestry estimates for both Afro-descendant communities and Native American tribes showed different means compared to other populations from the same region in Brazil (Table S3 and Table 1).

## Discussion

Several studies have pointed out the differences of genetic ancestry among the regions of Brazil (Callegari-Jacques *et al.*, 2003; Godinho *et al.*, 2008; Lins *et al.*, 2010; Manta *et al.*, 2013a). We performed an extensive review of the literature for a better understanding of the heterogeneity of the Brazilian population from different geographic regions and analyzed the influence of some factors, such as the molecular marker type and number of samples, in ancestry estimates. Nineteen states of Brazil were represented in this study and only seven were not included because studies about these states did not match our inclusion criteria or they did not have published information. We found that the mean proportions of European, African, and Native American ancestries in Brazil were 68.1%, 19.6%, and 11.6%, respectively. Prior to ours, Moura *et al.* (2015) performed a systematic review with meta-analysis of the genetic admixture studies of the Brazilian population compared to other countries in Latin America. Although that study included a lower number of populations from different Brazilian states (n=38, this study n=81), the authors found similar means of genetic ancestry (European – 62%, African – 21% and Native American – 17%).

A global overview of all studies showed a major European contribution across regions of the country. However, the distribution of the ancestral groups along the Brazilian territory did not occur homogeneously, differing significantly depending on the geographic region. This heterogeneity can be explained by the occupation history of each region. The Northeast was the cradle of Portuguese colonization, and it was there where they landed and established the great majority of African slaves (Holanda, 1995; Ribeiro, 1995; Schaan *et al.*, 2017), explaining the great African ancestry observed in this region. We identified that Bahia and Ceará had distinct profiles of genetic ancestries within the Northeast region. This result has suggested that Salvador and Jequié, both cities in Bahia state, might have preserved the African ancestry from the high number of slaves that were brought there. Salvador is considered the most African city in Brazil, with around 80% of Afro-descendants (Azevedo *et al.*, 1981; IBGE at https://ww2.ibge.gov.br/home/default.php). Moreover, the slaves arriving in Bahia were mainly originated from Nigeria, Ghana, and Benin, different from the slaves sent to other Brazilian States, who came mainly from Angola, Congo, and Mozambique (Verger, 1968; Kehdy *et al.*, 2015). However, it is not clear whether the actual genetic profile of the populations from Bahia and Ceará were influenced by this differential slave trade. In the mid-seventeenth century, the decline of the sugar economy in the Northeast Region and the discovery of gold and precious stones in areas of the actual state of Minas Gerais caused a great migration of Africans to the Southeast (Krieger *et al.*, 1965; Schwartz, 1988; Ribeiro, 1995). This migration caused a great genetic admixture in the southeastern populations, overlapping in the tri-plot with populations from the Northeast and South. In the North, due to the isolated geographic location and the Amazon forest, the occupation process occurred later and more effectively because of governmental development projects (Martine, 1990; de Castro *et al.*, 2007). This region has a high number of Native Americans, and due to social policies that encouraged marriage between white men and Native American women, this region has a great influence of the Native American genetic ancestry (Benchimol, 1999; Carneiro-da-Cunha, 1992; Batista dos Santos *et al.*, 1999). One of the highest proportions of Native Americans (38%) was observed in studies conducted in the Amazonas state (Manta *et al.*, 2013a; Kano *et al.*, 2016; 2018).

In the South there was an intense migratory flow of Europeans, especially in the 18th and 19th centuries (Holanda, 1995; Carvalho-Silva *et al.*, 2001; Marrero *et al.*, 2005; Pena, 2005; Pena *et al.*, 2011). In this region, the first group of colonizers consisting of Portuguese immigrants arrived in 1808, and later the arrival of Europeans for wage labor was encouraged by the government (Salzano and Freire-Maia, 1967; Ribeiro, 1995; Alves-Silva *et al.*, 2000; Marrero *et al.*, 2005; Pena *et al.*, 2011). This region was sparsely inhabited by Native Americans due to the attacks of Portuguese colonizers (Leite, 1996; Ramos, 2006; Ribeiro, 1995). In addition, the area received small numbers of African slaves (Levy, 1974). This immigration process explains why this region showed the most different proportions of genetic ancestries, with the highest contribution of European ancestry (81.8%) in Brazil.

For the Central-West region only five studies were selected, and further studies are needed to better understand the genetic makeup of this population. The occupation of this region occurred due to the expansion of the agricultural frontier and the construction of the city of Brasília in the 1970’s (Cunha, 2002, 2006). This region received migrants from all parts of Brazil, showing a strong overlap in the tri-plot with Southeast and Northeast populations.

The estimates of genetic ancestry depend on the selected molecular marker panel, method used to infer population admixture, and the sampling method. For this study, we selected only studies that used nuclear autosomal markers. We excluded the studies that used classical markers, published between the years 1957-2000, which are based indirectly on variation in the products of genes. Despite the unquestionable value of classical markers for human ancestry understanding, as summarized by Cavalli-Sforza *et al.* (1994), we chose to include only nuclear markers. These markers allow to infer the individual ancestry and are widely used in genetic association studies to control for statistical biases related to population stratification between cases and controls or as a strategy to map susceptibility alleles associated with diseases that differ between recently admixed populations (Seldin, 2007; Tian *et al.*, 2008). Uniparental genetic markers, despite being good lineage markers, the maternal lineage (mitochondrial inheritance), and the paternal lineage (Y chromosome) were also excluded because they represent only the contribution of one side of the ancestry history. Comparing the ancestry estimates for each geographic region, the only significant difference was in the average of European ancestry in the Northeast region estimated using INDELs compared to the estimate using SNPs.

Two studies carried out in the Amazonas state showed a highly consistent estimate of all three ancestries independent of the type of molecular markers (INDELs (Manta *et al.*, 2013a) or SNPs (Kano *et al.*, 2016). However, another study using STRs showed different estimates of ancestry for a population from the same state (Callegari-Jacques *et al.*, 2003). Half of the studies based on samples from same cities showed significant differences in ancestry estimates using distinct types of molecular markers. The difference in ancestry estimates was found between INDEL and SNP (e.g., Belem and Fortaleza), and between INDEL and STR (e.g., Manaus and Porto Velho). However, these comparisons must be viewed with caution, since studies using different sample of individuals from different cities or even from the same city may differ in the level of admixture. The influence of molecular marker panels in the estimates of ancestry is clearer in studies based on the same sample using different maker panels. For instance, Pedrosa (2006) showed different ancestry estimates for the same sample of individuals analyzed using three different panels of markers (8 STRs, 9 SNP/INDELs, and a combination of both). STR panel showed the most distinct results compared to the other two panels (Table S3). Teló (2010) also reported different ancestry estimates using INDELs and STRs for the same sample of individuals from Salvador/BA (Table 1). Microsatellite markers, frequently used in individual identification, as forensic markers used in all studies included in this review, were not able to detect differences among populations and, consequently, among ethnic groups (Barnholtz-Sloan *et al.*, 2005; Pedrosa, 2006). For microsatellites, it is not possible to claim that similar allelic frequencies in specific loci are directly related to common ancestries or share the same evolutionary history (Santos and Tyler-Smith, 1996). They are markers of fast evolution with high rates of mutation (Ellegren, 2004) with different allelic frequencies in admixture and in parental populations, distorting the ancestry profile of the population. On the other hand, SNPs and INDELs are markers of slow evolution (Stoneking, 2001; Weber *et al.*, 2002). Studies have highlighted that some microsatellites could be more informative than SNPs (Ruiz Linares, 1999; Pfaff *et al.*, 2004; Tang *et al.*, 2005). Therefore, more important than the type of marker is the selection of markers based on their selectivity for ancestral populations (Parra *et al.*, 2001).

An important aspect to be considered for genetic ancestry estimates is the minimum number of markers needed to determine the proportions of parental ancestry within a population. This analysis was possible only for studies based on samples from the same city using different numbers of the same type of marker. Here, only studies using INDELs were compared, without any significant difference between ancestry estimates and number of markers. Ding *et al.* (2011) showed that using the top 20 ranked informative markers accurately classified all ancestral populations. Lao *et al.* (2006) suggested that as few as 10 highly informative SNP markers were able to differentiate individuals from Africa, Europe, Asia, and America. Kosoy *et al.* (2009) also have shown that a few dozen well-selected markers have a similar power of discrimination to sets of larger numbers of random markers. Therefore, the number of markers is highly dependent of the informativeness of the panel as discussed above. The number of markers for ancestry estimates will also depend on the level of genetic differentiation of the admixture populations and the desired stringency of assignment (Baye *et al.*, 2011).

One of the factors of great importance for studies on population ancestry is the nature of the sample. Many studies performed the ancestry estimates using selected samples from hospitals, clinics, universities, diagnostic units, or specific areas of the city, which may not correctly represent the ancestral estimates of the entire population. Moreover, it is very difficult to obtain a truly random sample of the population to be studied. Here, we determined the mean of ancestry weighted by the number of samples to balance potential bias in the sampling. For instance, in the study of Kehdy *et al.* (2015) the sample from Salvador/BA was from poor areas of the city, with a strong bias for higher African ancestry than the city as a whole (Barreto *et al.*, 2006). Moreover, we did not identify any statistically significant correlation among the number of individuals in the studies performed from the same region or the same cities using the same type of molecular marker and the ancestry estimates. Therefore, the number of individuals per se did not seem to influence the ancestry estimates.

Studies that selected only a few ethnic subgroups of the population were excluded since they might not represent the ancestry estimates of the whole population, such as skin color subgroups studied by Pena *et al.* (2011). Similarly, some studies were excluded as they included relatives who may cause bias in ancestry estimates (Leite *et al.*, 2011; Giolo *et al.*, 2012). Moreover, studies of specific ethnic communities that have little contact with the dominant civilization, such as Native American tribes and communities of Afro-descendants originally formed by fugitive slaves, the Quilombos, were analyzed separately. The Native American tribes showed differences in estimated average ancestries compared to the Brazilian population. The Afro-descendant communities showed a highly diverse estimate of ancestry with significant differences only for the mean of African and European ancestries compared to the average of other Brazilian populations. This variability reflects the history of each community with different levels of admixture, and the ability of molecular markers to identify genetic ancestries in admixture population, as discussed above for the data from Pedrosa (2006).

The history of the formation of quilombos was quite variable across the country, with distinct levels of interaction with Europeans and Native Americans. The distribution of Native American tribes and Afro-descendants’ communities are asymmetrical over the Brazilian territory. Consistent with the highest African ancestry, the Northeast region also has the highest number of Afro-descendant communities (n=1,724). Moreover, the North region has the highest Native American ancestry and also higher number of Native American individuals (305,873 individuals - 37.4% of the total indigenous population in the country) (www.funai.gov.br/index.php/indios-no-brasil/so-sao).

The correlation between genetic ancestry and ethno-racial classification in Brazilian individuals was not the aim of this review. However, it is important to mention the recent study by Ruiz-Linares *et al.* (2014) that analyzed more than 7,000 individuals from Latin America including 1,594 Brazilians and showed a significant correlation between genetic ancestry and the self-perception of ancestry. Despite the correlation found, the authors suggested that some physical appearance traits and other factors, possibly non-biological factors, biased the self-perception of ancestry. Lima-Costa *et al.* (2015) also showed a significant correlation that is most consistent with the extremes of African proportions.

Several studies have pointed out high gene flow and genetic heterogeneity among regions of Brazil. In this study, we scanned the literature to obtain a robust review of the Brazilian population’s ancestry. In 81 populations included in this review there was a predominance of European ancestry, which reflects the process of colonization and the relatively recent European mass immigration to Brazil. In the general population, we identified a low contribution of Native American ancestry that may be due in part to the mass extermination of this population during the colonial period. In areas where slaves were widely used as cheap labor, as in the Northeast and Southeast regions, we found a greater African ancestry. Of importance, this review contributes for a broader understanding of the genetic makeup of the Brazilian population and the roles of marker types and sampling method in the genetic ancestry estimates.

## Supplementary material

The following online material is available for this article:

Table S1 Terms used in the manuscripts search in the BVS and PUBMED database/portal.

Table S2 Characterization of the 51 manuscripts included in the scoping review.

Table S3 Characterization of selected studies using partially isolated populations: Afro-descendants' communities and Native American tribes.

Figure S1 Ancestral estimates for populations from different states of each Brazilian region.
