# Supplementary material for: A systematic scoping review of the genetic ancestry of the Brazilian population
Source: Genet Mol Biol. 2019 Nov 14;42(3):495–508. doi: 10.1590/1678-4685-GMB-2018-0076 (PMC6905439; doi:10.1590/1678-4685-GMB-2018-0076)
Supplement: Supplementary file 1 [file 1415-4757-GMB-42-3-2018-0076-suppl1.pdf]

Supplementary Material to “A systematic scoping review of the genetic ancestry of the Brazilian population”

Table S1 - Terms used in the manuscripts search in the BVS and PUBMED database/portal.

Search date	Search engine	Number of publications retrieved	Search terms
01/10/2017	BVS	809	((((tw:(Genética OR Genetics OR "Marcadores Genéticos" OR "Genetic Markers" OR "Marcadores Cromossômicos" OR "Marcadores de DNA" OR "Marcadores Cromosómicos" OR "Marcadores de ADN" OR "Genetic Marker" OR "Marker, Genetic" OR "Chromosome Marker" OR "DNA Marker" OR "Marker, Chromosome" OR "Marker, DNA" OR "Markers, Chromosome" OR "Chromosome Markers" OR "Markers, Genetic" OR "DNA Markers" OR "Markers, DNA"))) AND ((tw:("Grupos Populacionais" OR "Grupos de Población" OR "Population Groups" OR "Grupo com Ancestrais do Continente Africano" OR "Grupo de Ascendencia Continental Africana" OR "African Continental Ancestry Group" OR "Grupo com Ancestrais do Continente Europeu" OR "Grupo de Ascendencia Continental Europea" OR "European Continental Ancestry Group" OR "População Indígena" OR "Población Indígena" OR "Indigenous Population" OR "Grupo Populacional" OR "Grupos de População" OR "Grupo de Ancestralidade no Continente Africano" OR "Grupo com Ancestrais Africanos Continentais" OR "Grupo de Ascendência Africana Continental" OR "Grupo de Ascendência Continental Africana" OR "População Negra" OR "Grupo de Ancestralidade no Continente Europeu" OR "Grupo com Ancestrais Europeus Continentais" OR "Grupo de Ascendência Continental Europeia" OR "Grupo de Ascendência Europeia Continental" OR "Raça Caucasoide" OR "Camponeses Indígenas" OR "Comunidades Autóctones" OR "Populações Autóctones" OR "Comunidades Indígenas" OR "Povos Nativos" OR "Povos Indígenas" OR "Indígenas Nativos" OR "Tribos Nativas" OR Tapeba OR Tapebas OR "Grupo Poblacional" OR "Grupos Poblacionales" OR "Grupo de Población" OR "Grupo de Ancestro Africano Continental" OR "Población Negra" OR "Grupo de Ancestro Europeo Continental" OR "Raza Caucasoide" OR "Campesinos Indígenas" OR "Comunidades Autóctonas" OR "Poblaciones Autóctonas" OR "Comunidades Indígenas" OR "Pueblos Nativos" OR "Indigenous Population" OR "Native-Born" OR "Natives Tribes" OR "Group, Population" OR "Groups, Population" OR "Indigenous Populations" OR "Native Born" OR "Population Group" OR "Population, Indigenous" OR "Populations, Indigenous" OR "Negroid Race" OR "Negroid Races" OR "Race, Negroid" OR "Races, Negroid" OR "Caucasian Race" OR "Caucasoid Race" OR "Caucasian Races" OR "Caucasoid Races" OR "Race, Caucasian" OR "Race, Caucasoid" OR "Races, Caucasian" OR "Races, Caucasoid")))) AND (la:("en" OR "es" OR "pt"))) AND (Brasil OR Brazil OR Brasileiro OR Brazilian)
02/10/2017	Pubmed	88	((Brazil OR Brazilian)) AND ((((("Population Groups"[Title/Abstract] OR "African Continental Ancestry Group"[Title/Abstract] OR "European Continental Ancestry Group"[Title/Abstract] OR "Indigenous Population"[Title/Abstract] OR "Native-Born"[Title/Abstract] OR "Natives Tribes"[Title/Abstract] OR "Group, Population"[Title/Abstract] OR "Groups, Population"[Title/Abstract] OR "Indigenous Populations"[Title/Abstract] OR "Native Born"[Title/Abstract] OR "Population Group"[Title/Abstract] OR "Population, Indigenous"[Title/Abstract] OR "Populations, Indigenous"[Title/Abstract] OR "Negroid Race"[Title/Abstract] OR "Negroid Races"[Title/Abstract] OR "Race, Negroid"[Title/Abstract] OR "Races, Negroid"[Title/Abstract] OR "Caucasian Race"[Title/Abstract] OR "Caucasoid Race"[Title/Abstract] OR "Caucasian Races"[Title/Abstract] OR "Caucasoid Races"[Title/Abstract] OR "Race, Caucasian"[Title/Abstract] OR "Race, Caucasoid"[Title/Abstract] OR "Races, Caucasian"[Title/Abstract] OR "Races, Caucasoid"[Title/Abstract])) OR ((("Population Groups"[Mesh:noexp]) OR "African Continental Ancestry Group"[Mesh:noexp]) OR "European Continental Ancestry Group"[Mesh:noexp]))) AND ((("Genetics"[Title/Abstract] OR "Genetic Markers"[Title/Abstract] OR "Genetic Marker"[Title/Abstract] OR "Marker, Genetic"[Title/Abstract] OR "Chromosome Marker"[Title/Abstract] OR "DNA Marker"[Title/Abstract] OR "Marker, Chromosome"[Title/Abstract] OR "Marker, DNA"[Title/Abstract] OR "Markers, Chromosome"[Title/Abstract] OR "Chromosome Markers"[Title/Abstract] OR "Markers, Genetic"[Title/Abstract] OR "DNA Markers"[Title/Abstract] OR "Markers, DNA"[Title/Abstract])) OR (("Genetics"[Mesh:noexp]) OR "Genetic Markers"[Mesh:noexp]))) AND (English[lang] OR Portuguese[lang] OR Spanish[lang]))
