# Supplementary material for: A systematic scoping review of the genetic ancestry of the Brazilian population
Source: Genet Mol Biol. 2019 Nov 14;42(3):495–508. doi: 10.1590/1678-4685-GMB-2018-0076 (PMC6905439; doi:10.1590/1678-4685-GMB-2018-0076)
Supplement: Supplementary file 2 [file 1415-4757-GMB-42-3-2018-0076-suppl2.pdf]

Supplementary Material to "A systematic scoping review of the genetic ancestry of the Brazilian population"

Table S2 - Characterization of the 51 manuscripts included in the scoping review.

Region	State	City	N	EUR	AFR	NAM	Number of markers	Molecular marker type	Reference
Center-West	Distrito Federal	Brasília	200	77	14	9	28	SNP	4
			168	61	23	16	15	Alu/SNP/INDEL	2
			412	57	29	14	12	SNP	3
		Taguatinga	189	63	25	12	13	SNP	1
	Mato Grosso do sul	NI	84	59	26	15	46	INDEL	5
North	Para	Belém*	167	40	30	30	8	SNP	6
			203	70	11	19	40	INDEL	7
			226	54	16	30	46	INDEL	8
			196	61	12	27	48	INDEL	9
			125	50	21	29	48	INDEL	10
			127	52	20	28	48	INDEL	11
		NI	210	45	23	32	62	INDEL	12
		Santarém	90	60	11	29	40	INDEL	13
		Goianésia do Pará	273	44	32	24	48	INDEL	14
	Amapá	Macapá[#]	130	50	29	21	48	INDEL	15
			307	46	19	35	12	STR	16
	Amazonas	Manaus*	252	71	12	17	12	STR	17
			42	46	16	38	46	INDEL	5
		Rio Pardo	340	44	18	38	48	SNP	18
	Rondônia	Porto Velho*	122	60	30	11	4	STR	19

Region	State	City	N	EUR	AFR	NAM	Number of markers	Molecular marker type	Reference
			282	54	18	28	62	SNP/INDEL	20
Northeast	Ceará	Barbalha	60	68	19	13	40	INDEL	13
		Fortaleza*	216	71	13	16	40	INDEL	13
			616	49	16	35	237	SNP	21
			110	53	22	25	199654	SNP	22
			276	54	22	24	199654	SNP	22
			336	52	23	25	199654	SNP	22
			95	55	22	23	199654	SNP	22
			63	54	21	25	199654	SNP	22
	Bahia	Salvador#	206	80	18	2	40	INDEL	23
			852	63	34	3	40	INDEL	24
			1309	43	51	6	331790	SNP	25
			511	51	40	9	237	SNP	21
			203	45	45	10	9	INDEL*	26
			203	59	33	8	8	STR*	26
			289	44	49	7	7	Alu/INDEL/RFLP	27
			1286	36	49	15	10	Alu/INDEL/SNP	28
		Ilheus	109	61	33	6	11	Alu/INDEL/SNP	29
			147	61	30	9	40	INDEL	7
		Jequie	20	44	42	11	8	SNP	30
	Alagoas	Maceio	120	67	19	14	40	INDEL	13
		NI	104	55	27	19	46	INDEL	5
	Maranhão	São Luís	177	42	19	39	4	STR	31
	Piauí	NI	204	60	22	18	46	INDEL	32
	Pernambuco	NI	133	57	28	15	46	INDEL	5

Region	State	City	N	EUR	AFR	NAM	Number of markers	Molecular marker type	Reference
		Recife	192	60	23	17	12	SNP	33
South	Rio Grande do Sul	Porto Alegre#	226	82	11	7	12	STR	17
			6782	85	4	9	48	INDEL	34
			189	78	13	9	40	INDEL	7
		Pelotas	3736	76	16	8	331790	SNP	25
		NI	81	95	1	4	48	INDEL	9
		NI	23	73	14	13	46	INDEL	5
	Paraná	NI	21	71	17	12	46	INDEL	5
	Santa Catarina	NI	20	80	11	9	46	INDEL	5
Southeast	Espirito Santo	NI	92	74	13	13	46	INDEL	5
	Minas Gerais	Belo Horizonte*	234	65	34	1	13	STR	35
			90	76	15	10	40	INDEL	36
		Alfenas	299	89	9	2	40	INDEL	23
			459	87	11	2	40	INDEL	37
		NI	88	59	29	12	46	INDEL	5
		NI	291	58	34	4	54	SNP/INDEL	38
		Manhuaçu	30	63	27	9	14	SNP	39
		Montes Claros	24	54	41	5	14	SNP	39
		Ouro Preto	189	50	33	16	15	SNP	40
		Bambui	1442	79	14	7	331790	SNP	25

Region	State	City	N	EUR	AFR	NAM	Number of markers	Molecular marker type	Reference
	São Paulo	NI	49	63	25	12	46	INDEL	5
		Araraquara	403	76	18	6	15	STR	41
		São Paulo#	326	71	19	10	40	INDEL	37
			145	73	10	7	48	SNP	42
			362	58	33	9	101348	SNP	43
			503	58	28	14	48	INDEL	44
			1010	75	19	6	31	SNP	45
		Botucatu	593	80	8	7	61	INDEL	46
			390	67	16	12	61	INDEL	47
		Campinas	109	73	20	7	12	STR	17
		Ribeirão Preto	448	86	9	4	48	INDEL	48
	Rio de Janeiro	Rio de Janeiro	116	65	23	12	40	INDEL	13
			413	55	31	14	46	INDEL	49
			264	74	19	7	40	INDEL	7
			87	74	20	7	40	INDEL	50
		NI	335	65	28	7	40	INDEL	51

*Significant differences comparing ancestry estimates by different molecular markers (Spearman's test, $p<0.05$). #Not significant differences comparing the two sets of markers. References: 1. Lins *et al*, 2011a; 2. Gontijo *et al*., 2014, 3. Bened Morais *et al*., 2012; 4. Lins *et al*., 2011b; 5. Manta *et al*., 2013b; 6. Cardoso *et al*., 2014, 7. Pena *et al*., 2011; 8. Pereira *et al*., 2012; 9.Santos *et al*., 2010; 10.Carvalho *et al*., 2015; 11. Vieira *et al*., 2015; 12. Vieira-Machado *et al*., 2016; 13. Brito *et al*., 2011; 14. Cassiano *et al*., 2015; 15. Francez *et al*., 2011; 16. Francez *et al*., 2012; 17. Callegari-Jacques *et al*., 2003; 18. Kano *et al*., 2016; 19. França, 2005; 20. Tarazona-Santos *et al*., 2011; 21. Silva *et al*., 2015; 22. Mychaleckyj *et al*., 2017; 23. Aquino *et al*., 2014; 24. do Rego Borges *et al*., 2015; 25. Kehdy *et al*., 2015; 26. Teló, 2010; 27. Felix *et al*., 2010; 28. Machado, 2008; 29. Oliveira *et al*., 2016; 30. Nascimento *et al*., 2016; 31. Ferreira *et al*., 2002; 32. Lopes *et al*., 2014; 33. Coelho *et al*., 2015; 34. Wagner *et al*., 2016; 35. Scliar *et al*., 2009; 36. Rolim *et al*., 2016; 37. Brito *et al*., 2012; 38. Silva *et al*., 2011; 39. Silva *et al*., 2010; 40. Queiroz *et al*., 2013; 41. Martins *et al*., 2011; 42. Nastri *et al*., 2016; 43. Bermardez-Pereira *et al*., 2016; 44. Cardena *et al*., 2013; 45. Guindalini, 2010; 46. D'Éllia *et al*., 2017; 47. Ramos *et al*., 2016; 48. Souza *et al*., 2015; 49. Manta *et al*., 2013a 52. Santos *et al*., 2009; 51. Suarez-Kurtz *et al*., 2007.

References exclusively cited in this table:

Felix GES, Abe-Sandes K, Bonfim TM, Bendicho MT, Cisneiros P, Guedes R, Brandão CJF, Torres AJL, Brites C, Netto EM *et al*. (2010) Ancestry informative markers and complete blood count parameters in Brazilian blood donors. Rev Bras Hematol Hemoter 32:282-285.

Machado TMB (2008) Ancestralidade em Salvador – BA. M. Sc. Thesis, Fundação Oswaldo Cruz, Centro de Pesquisas Gonçalo Moniz, Salvador, 86 p.

Oliveira JS, Ferreira RS, Santos LM, Marin LJ, Corrêa RX, Luizon MR, Simões AL, Gadelha SR and Sousa SM (2016) Self-declared ethnicity and genomic ancestry in prostate cancer patients from Brazil. Genet Mol Res 15:15048769.

Tarazona-Santos E, Castilho L, Amaral DR, Costa DC, Furlani NG, Zuccherato LW, Machado M, Reid ME, Zalis MG, Rossit AR *et al*. (2011) Population genetics of GYPB and association study between GYPB*S/s polymorphism and susceptibility to *P. falciparum* infection in the Brazilian Amazon. PLoS One 6:e16123.
