# Supplementary material for: A systematic scoping review of the genetic ancestry of the Brazilian population
Source: Genet Mol Biol. 2019 Nov 14;42(3):495–508. doi: 10.1590/1678-4685-GMB-2018-0076 (PMC6905439; doi:10.1590/1678-4685-GMB-2018-0076)
Supplement: Supplementary file 3 [file 1415-4757-GMB-42-3-2018-0076-suppl3.pdf]

Supplementary Material to "A systematic scoping review of the genetic ancestry of the Brazilian population"

Table S3 - Characterization of selected studies performed using partially isolated populations: Afro-descendants communities and Native American tribes.

Region	State	City	Blood colletion time	N	EUR	AFR	NAM	Number of markers	Marker type	Reference
**Afro-descendant communities (Quilombos)**										
Center-West	Goiás	Kalunga	2001-2002	72	4.0**	89.0**	7.0	8	STR	52
			2001-2002	72	30.0**	61.0**	9.0	9	SNP/INDEL	52
			2001-2002	72	30.0**	61.0**	8.0	17	SNP/INDEL/STR	52
	Mean of the region			216	**21.3 ± 15.0***	**70.3 ± 16.2***	**8.0 ± 1.0***			
North	Pará	Pacoval	ND	30	11.0	51.0	38.0	6	VNTR/STR	53
			ND	21	30.0	38.0	32.0	48	INDEL	54
		Marajó	ND	48	28.0	41.0	31.0	48	INDEL	54
		Pitimandeua	ND	64	32.0	49.0	19.0	48	INDEL	54
		Trombetas	ND	40	20.0	53.0	28.0	48	INDEL	54
	Amapá	Curiau	ND	34	24.0	42.0	33.0	6	VNTR/STR	53
			ND	43	22.0	58.0	20.0	48	INDEL	54
		Mazagão	ND	45	41.0	38.0	21.0	48	INDEL	54
	Mean of the region			358	**26.0 ± 9.0***	**46.2 ± 7.5***	**27.7 ± 7.0**			
Northeast	Bahia	Rio das Rãs	1998	278	38.0	52.0	10.0	10	SNP	55
			1998	102	19.0	78.0**	3.0	8	STR	52
			1998	102	31.0	61.0**	8.0	9	SNP/INDEL	52
			1998	102	32.0	62.0**	7.0	17	SNP/INDEL/STR	52
		Sacutiaba	1998	70	55.0	43.0	2.0	10	SNP	55
			1998	30	74.0**	26.0**	0	8	STR	52
			1998	30	43.0**	54.0**	2.0	9	SNP/INDEL	52
			1998	30	40.0**	43.0**	16.0	17	SNP/INDEL/STR	52
	Piauí	Mimbo	1993	176	17.0	61.0	22.0	10	blood proteins /STR	56
		Sitio Velho	1993	185	12.0	72.0	16.0	10	blood proteins / STR	56
	Maranhão	Pontal	ND	33	26.0	61.0	13.0	48	INDEL	54

Region	State	City	Blood colletion time	N	EUR	AFR	NAM	Number of markers	Marker type	Reference
	Sergipe	Mocambo	1998	77	55.0**	34.0	11.0	8	STR	52
			1998	77	38.0**	43.0	18.0	9	SNP/INDEL	52
			1998	77	42.0**	51.0	6.0	17	SNP/INDEL/STR	52
			1998	171	35.0	46.0	19.0	10	SNP	55
	Mean of the region			1540	**37.1 ± 16.1***	**52.5 ± 13.9***	**10.2 ± 6.9***			
Southeast	São Paulo	Região do Vale da Ribeira	ND	307	39.0	40.0	21.0	48	INDEL	57
	Minas Gerais	Marinhos	ND	60	37.0	59.0	4.0	14	STR	35
	Mean of the region			367	**38.0 ± 14.1**	**49.5 ± 13.4**	**12.5 ± 12.0**			
Mean of the country				2448	**32.7 ± 14.6***	**52.4 ± 13.7***	**14.8 ± 10.5**			
**Native American tribes**										
Center-West	Mato Grosso do Sul	Terena	ND	42	13.0	9.0	78.0	46	INDEL	5
North	Amazonas	Santa Isabel	ND	100	17.0	7.0	76.0	46	INDEL	5
Mean of the country					**15.0 ± 2.8**	**8.0 ± 1.4**	**77.0 ± 1.4**			

*Significantly different from the mean of non-partially isolated Brazilian populations (Table 1) (Mann-Whitney U test, $p<0.001$). **Significantly different in the same samples analyzed using different markers (Test Z, $p<0.05$, according to Pedrosa, 2006). ND: not determined. INDEL - insertion/deletion makers, STR - short tandem repeat , VNTR - Variable number of tandem repeat, SNP - single nucleotide polymorphism. References: 5. Manta *et al*., 2013b; 52. Pedrosa, 2006; 53. Vallinoto *et al*., 2003; 54. Lopes Maciel *et al*., 2011; 55. Amorim *et al*., 2011; 56. Arpini-Sampaio *et al*., 1999; 57. Kimura *et al*., 2012; 35. Schiar *et al*., 2009.

References exclusively cited in this table

Amorim CE, Gontijo CC and Falcão-Alencar G AL (2011) Migration in Afro-Brazilian rural communities: Crossing demographic and genetic data. Hum Biol 83:509-521.

Arpini-Sampaio Z, Costa MC, Melo AA, Carvalho MF, Deus MS and Simões AL (1999) Genetic polymorphisms and ethnic admixture in African-derived black communities of northeastern Brazil. Hum Biol 71:69-85.

Kimura L, Ribeiro-Rodrigues EM, Auricchio MTM, Vicente JP, Santos SEB and Mingroni-Netto RC (2013) Genomic ancestry of rural African-derived populations from Southeastern Brazil. Am J Hum Biol 25:35-4.

Maciel LLGL, Rodrigues EMR, Santos NPC, Santos AR, Guerreiro JF and Santos S (2011) Afro-derived Amazonian populations: Inferring continental ancestry and population substructure. Hum Biol 83:627-636.

Vallinoto IMC,Vallinoto ACR, Valente CMD AL and Guerreiro JF (2003) Allele frequency distributions of six hypervariable loci (D1S80, APOB, D4S43, vW1, F13A and DYS19) in two African-Brazilian communities from the Amazon region. Genet Mol Biol 26:235-240.
