# Supplementary material for: A systematic scoping review of the genetic ancestry of the Brazilian population
Source: Genet Mol Biol. 2019 Nov 14;42(3):495–508. doi: 10.1590/1678-4685-GMB-2018-0076 (PMC6905439; doi:10.1590/1678-4685-GMB-2018-0076)
Supplement: Supplementary file 4 [file 1415-4757-GMB-42-3-2018-0076-suppl4.pdf]

Supplementary Material to "A systematic scoping review of the genetic ancestry of the Brazilian population"

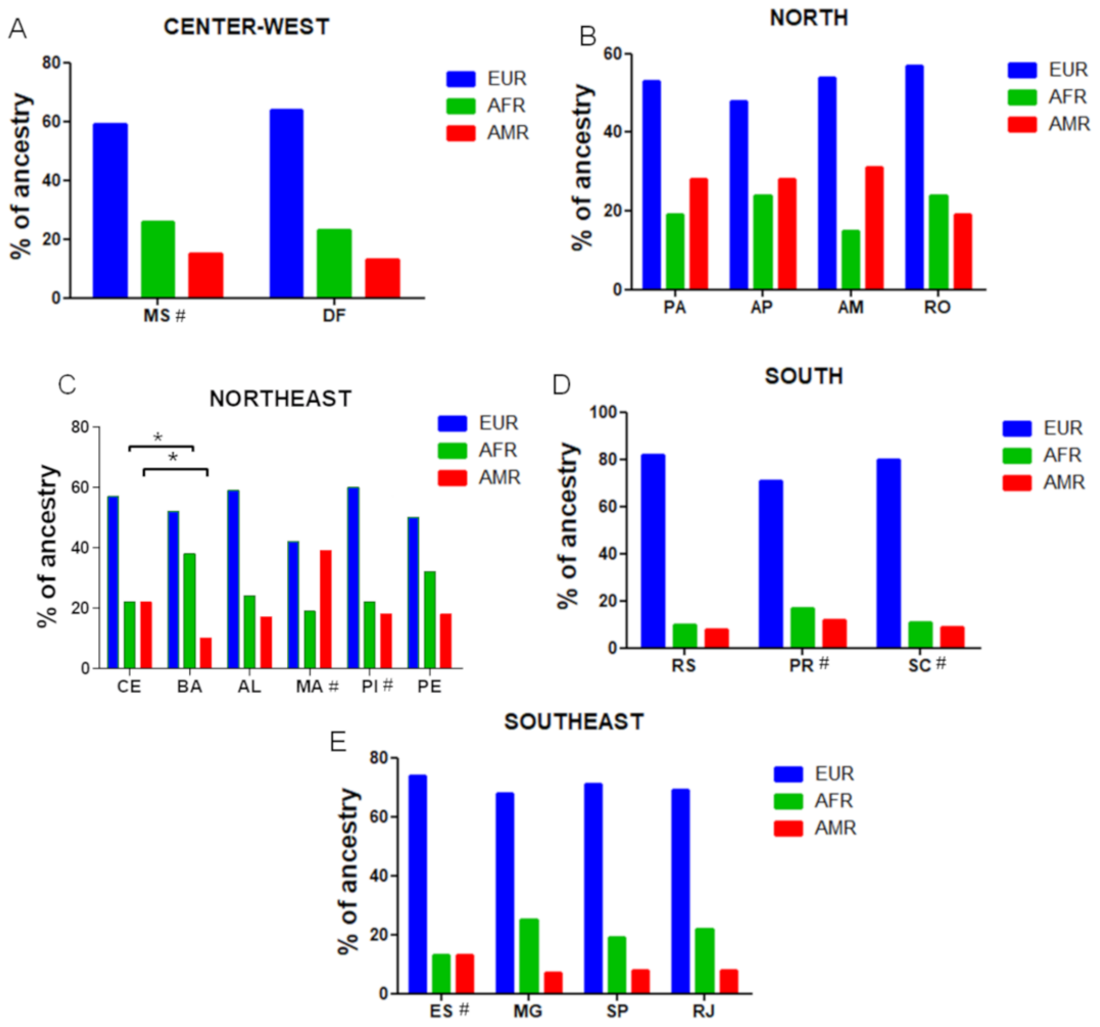

Figure S1 - Ancestral estimates for populations from different states of each Brazilian region. The graphs represent the median of genetic ancestry for all studies performed with individuals from each state. Asterisks indicate statistical differences between groups (Kruskal-Wallis test, $p<0.005$).

#Indicates the data from only one study.
